# Spent Mushroom Substrate-Derived Biochar and Its Applications in Modern Agricultural Systems: An Extensive Overview

**DOI:** 10.3390/life15020317

**Published:** 2025-02-18

**Authors:** Worawoot Aiduang, Kritsana Jatuwong, Tanongkiat Kiatsiriroat, Wassana Kamopas, Pimsiri Tiyayon, Rotsukon Jawana, Orlavanh Xayyavong, Saisamorn Lumyong

**Affiliations:** 1 Office of Research Administration, Chiang Mai University, Chiang Mai 50200, Thailand; worawoot.aiduang@cmu.ac.th (W.A.); kritsana.ja@cmu.ac.th (K.J.); 2 Department of Biology, Faculty of Science, Chiang Mai University, Chiang Mai 50200, Thailand; xorlavanh@yahoo.com; 3 Department of Mechanical Engineering, Faculty of Engineering, Chiang Mai University, Chiang Mai 50200, Thailand; tanongkiat_k@yahoo.com; 4 Multidisciplinary Research Institute, Chiang Mai University, Chiang Mai 50200, Thailand; wassana.kamopas@cmu.ac.th; 5 School of Agricultural Resources, Chulalongkorn University, Bangkok 10330, Thailand; pimsiri.t@chula.ac.th; 6 Energy Research and Development Institute-Nakornping, Chiang Mai University, Chiang Mai 50200, Thailand; rotsukon.j@cmu.ac.th; 7 Center of Excellence in Microbial Diversity and Sustainable Utilization, Chiang Mai University, Chiang Mai 50200, Thailand; 8 Academy of Science, The Royal Society of Thailand, Bangkok 10300, Thailand

**Keywords:** SMS biochar potential, carbon sequestration, soil amendment, sustainable agriculture, sustainable development goals (SDGs) 2 and 15

## Abstract

Spent mushroom substrate (SMS), a nutrient-dense byproduct of mushroom cultivation, has emerged as a promising feedstock for biochar production, offering a sustainable solution to modern agricultural and environmental challenges. This review explores SMS properties, its conversion into biochar, and its various applications. Due to its lignocellulosic structure, high organic matter (OM), and essential nutrients, SMS is ideal for pyrolysis, a process that enhances biochar’s porosity, nutrient retention, and carbon stability. These properties improve soil fertility, water retention, microbial activity, and plant growth while also contributing to climate change mitigation through carbon sequestration. SMS-derived biochar stands out for its superior benefits, including a balanced pH, a rich nutrient profile, and the ability to adsorb heavy metals, which mitigates soil and water contamination and minimizes toxic risks in the food chain. By enhancing soil structure, nutrient cycling, and moisture retention, SMS-derived biochar supports sustainable farming practices that reduce chemical fertilizer use and boost climate resilience. Beyond soil applications, SMS-derived biochar is effective in wastewater treatment, mitigating plant diseases, and improving mushroom cultivation substrates, thereby enhancing mycelial growth and productivity. Economically, it is a cost-effective alternative due to the abundant availability and inexpensive nature of SMS. Nevertheless, challenges still exist, particularly in optimizing production methods and ensuring consistency in biochar properties, influenced by variations in pyrolysis conditions and SMS types. Advances in production technology and sustainable practices are vital for scaling up SMS-derived biochar production. This paper emphasizes the transformative potential of SMS-derived biochar, advocating for its integration into circular economy frameworks and sustainable agricultural systems. Recommendations for future research and policy support are provided to maximize the ecological and economic benefits of SMS-derived biochar, fostering its widespread adoption in global agricultural and environmental strategies.

## 1. Introduction

SMS, a byproduct of mushroom cultivation, represents both a valuable resource and a waste management challenge in modern agricultural systems [1]. With an organic-rich composition and a nutrient profile often enriched with nitrogen (N), phosphorus (P), potassium (K) as well as other minerals (calcium (Ca), magnesium (Mg), sulfur (S), iron (Fe), manganese (Mn), zinc (Zn), copper (Cu), and sodium (Na) [1,2], SMS has garnered interest for its potential as a raw material in biochar production. However, given the extensive global production of mushrooms (estimated at approximately 48.34 million metric tons), particularly in the top 10 mushroom-producing countries in the world, including China, Italy, the United States of America, the Netherlands, Poland, Spain, France, Iran, Canada, and the United Kingdom, SMS disposal presents considerable environmental challenges [3]. As a significant byproduct of mushroom farming, SMS is frequently considered waste. If improperly managed, it can contribute to greenhouse gas emissions, leach nutrients into groundwater, and accumulate as waste, resulting in environmental hazards through the development of pathogenic microflora and the spread of fungal diseases. It also increases eutrophication and adds to the financial, logistical, and ecological burdens for mushroom farms [4,5]. Crucially, unsuitable disposal can result in disagreeable smells and draw insects, causing additional issues for the environment and community wellness [6]. Despite these challenges, SMS holds considerable potential as a valuable feedstock and carbon source, particularly for biochar production, which offers a sustainable solution to waste disposal while providing numerous agricultural benefits. Converting SMS into biochar not only addresses these environmental concerns but also yields a material with wide-ranging agricultural benefits, aligning with the principles of sustainable and circular economy practices [7,8,9].

Biochar, a form of carbon-rich material produced through pyrolysis, has shown immense promise for soil health, pollutant remediation, and water management, improves material sustainability, and even supports urban agriculture and green infrastructure [10]. When applied to agricultural soils, SMS-derived biochar can improve soil fertility, water retention, and carbon sequestration, making it a valuable asset in sustainable farming practices and modern agricultural systems [7]. The pyrolysis process, which heats organic material in low-oxygen conditions, allows for SMS to be transformed into a stable carbon form that is resistant to decomposition, locking carbon into the soil for extended periods [10,11]. Optimal pyrolysis conditions for SMS-derived biochar production involve carefully controlled temperatures and residence times, which affect the physicochemical properties of the resulting biochar [11,12]. This thermochemical conversion process requires energy and technical considerations; yet, it has proven to be economically viable for agricultural use, particularly when offset by the benefits to soil and crop productivity [7,13,14].

The physicochemical properties of SMS-derived biochar, including high porosity and surface area, pore size distribution, high carbon content, as well as nutrient and moisture retention capabilities, make it particularly effective in various agricultural applications [13,14]. Its chemical characteristics, such as pH, cation exchange capacity (CEC), and the potential for heavy metal adsorption, distinguish it from biochars derived from other organic wastes, positioning it as a robust soil amendment for multiple agricultural purposes [13,14,15]. SMS-derived biochar’s stability and persistence in the soil enable it to improve soil structure and nutrient availability, thus enhancing plant growth, yield, and biochemical response, while simultaneously resulting in reduced incidence of disease [7,16,17]. Additionally, the biochar’s ability to adsorb heavy metals and organic pollutants opens opportunities for its use in agricultural wastewater treatment, protecting water resources from contamination by pesticides [16].

Currently, SMS-derived biochar’s applications extend beyond soil health and include carbon sequestration, nutrient retention, improved plant growth and biochemical response, and even an enhancement of mushroom production systems [1,7,14,16,18]. Moreover, several previous studies have indicated that several biochars offer a natural approach to plant disease management [17,19]. This presents a good guideline for further utilization of SMS-derived biochar in the future. Several comparative studies reveal that SMS-derived biochar performs similarly or even superiorly to traditional biochars made from wood or agricultural residues, largely due to SMS’s unique nutrient profile and structural properties [14,20]. This performance, coupled with its abundance and low cost, supports SMS-derived biochar’s environmental and economic viability as an amendment in modern farming systems [14,15,21].

Despite its potential, challenges remain in standardizing SMS-derived biochar production and addressing environmental concerns [22]. The influence of biochar on soil microbial communities, potential contaminants, and regulatory needs are areas that require further exploration to maximize the safety and efficacy of SMS-derived biochar in agricultural systems. Moreover, long-term studies are essential to understand its persistence and impact on soil ecology over time. Therefore, this review comprehensively examines SMS-derived biochar’s properties, production methods, and diverse applications in modern agriculture, exploring its potential benefits, limitations, and future directions. We also investigate novel opportunities in precision agriculture, water management, and pollution control, positioning SMS-derived biochar as a versatile and impactful component in sustainable agricultural practices.

## 2. Properties of Spent Mushroom Substrate

### 2.1. Overview of the Composition and Nutritional Profile of SMS

SMS is a nutrient-rich organic byproduct generated after harvesting edible and medicinal mushrooms, including widely cultivated genera such as *Agaricus*, *Auricularia*, *Coprinus*, *Flammulina*, *Ganoderma*, *Hericium*, *Lentinula*, *Lentinus*, *Pleurotus*, and *Tremella* [7,12,13,16,20,23,24,25,26]. This byproduct is composed of residual mycelium, lignocellulosic compounds (lignin: 25–39.8%, hemicellulose: 19–34%, and cellulose: 38–48.7%), bioactive compounds, fibers, proteins, non-cell-wall carbohydrates, and essential minerals derived from agricultural residues such as sawdust, corn cob, cottonseed hull, straw, and wood chips, along with amendments like gypsum, lime, and peat [1,6,27,28,29].

Each SMS batch offers unique physicochemical properties, enriched with both macronutrients and micronutrients essential for plant growth (Table 1). For example, SMS holds high OM (58.97 to 92.73%) and vital nutrients like N (0.46–2.72%), P (0.02–0.84%), and K (0.02–2.40%). It is also a rich source of micronutrients such as Ca (0.52–10.92%), Mg (0.09–0.91%), S (0.2–1.6%), Fe (7.60–920 mg·kg^−1^), Mn (100–1600 mg·kg^−1^), Zn (26–220 mg·kg^−1^), Cu (10.80–79.67 mg·kg^−1^), and Na (433.33–6693.33 mg·kg^−1^), further enhancing its effectiveness as a soil amendment.

Notably, SMS has excellent physical properties, particularly its exceptional WHC, which ranges from 231.39 to 699.68%, significantly higher than traditional growing media like peat (~209.96%) or mixed plant growth media (~75.23%) [24]. This superior water retention capacity is invaluable for agricultural applications. Moreover, the residual fungal components in SMS contribute to a rich microbial profile, promoting soil biodiversity [31]. Its organic carbon (OC) and C/N ratio, ranging from 23.40 to 54.30% and 11.50:1 to 92.60:1, respectively, depending on the original substrate, makes it ideal for composting and biochar production. Similarly, its pH range (3.79–8.05) and EC (0.88–6.60 dS m^−1^) demonstrate its versatility across different soil types and crops. As a sustainable and non-toxic resource, SMS aligns with organic farming practices and supports the global movement toward sustainable agriculture, helping to foster more resilient and productive ecosystems [6].

### 2.2. Benefits of Biochar Conversion

SMS offers several advantages as a biochar feedstock, making it a valuable resource for sustainable biochar production [32]. As a material rich in lignocellulose, SMS has potential carbon sources that enable efficient carbonization during the pyrolysis process, resulting in its stable high-quality biochar [6,33]. This carbon-rich composition makes SMS-derived biochar an effective medium for carbon sequestration in soils or other growing substrates and could potentially mitigate climate change [34].

In addition, SMS contains a range of essential primary and secondary macronutrients, which are largely preserved during conversion to biochar. This nutrient retention enhances its role as a soil amendment by improving soil fertility and promoting plant growth when applied to agricultural systems [20,35,36]. In mushroom cultivation, studies also indicate that incorporating SMS-derived biochar into growing substrates can enhance mycelial growth and overall yield, demonstrating its utility across agricultural practices [14,20,36]. The physical properties of SMS, such as its natural porosity and high surface area, are maintained when it is converted to biochar, providing an ideal structure for water retention and fostering beneficial microbial habitats in soils [14,36]. Moreover, producing biochar from SMS addresses the environmental challenges of SMS disposal, transforming agricultural waste into a valuable byproduct and adding significant value to mushroom cultivation residues [6,27]. These combined benefits highlight SMS as an effective and sustainable feedstock for biochar, supporting both agricultural productivity and environmental health.

### 2.3. Environmental Concerns and Potential Impacts of SMS Disposal on the Ecosystem

The disposal of SMS presents several environmental challenges that can lead to significant ecological impacts [6]. As a major byproduct of mushroom cultivation, SMS is often regarded as waste, resulting in large quantities accumulating on farms or in landfills [37]. This accumulation can lead to various issues, such as the leaching of nutrients into water systems, which contributes to eutrophication. This process promotes harmful algal blooms that deplete oxygen levels in water environments, adversely affecting aquatic life and disrupting ecosystems [31,38]. Additionally, improper disposal can lead to unpleasant odors and attract pests, creating further environmental and public health concerns [6].

Additionally, SMS is rich in organic matter, and if not managed properly, it can undergo anaerobic decomposition, releasing potent greenhouse gases like methane (CH_4_), nitrous oxide (N_2_O), ammonia (NH_3_), and nitrate (NO^3−^), which have a far greater impact on climate change than carbon dioxide [37,39]. Without effective management practices, the environmental footprint of SMS can be considerable, highlighting the urgent need for sustainable waste management solutions [27]. Converting SMS into biochar, compost, or other value-added products offers a way to address these environmental challenges, transforming waste into a sustainable resource that can enhance agricultural practices and promote environmental health [6,27,28].

## 3. Production of Biochar from Spent Mushroom Substrate

### 3.1. Pyrolysis Process

Pyrolysis is a thermochemical process that converts biomass into biochar by heating it under controlled oxygen-limited conditions. As pyrolysis occurs at varying temperatures, the surface structure of the obtained biochar can change, influenced by the additional breakdown of lignin and other organic components in the feedstock [40]. The process generally operates within a temperature range of 300 to 1300 °C, where the medium (300–800 °C) and high temperatures (800–1300 °C) are tailored to optimize biochar properties depending on the desired application [41,42,43]. For SMS-derived biochar, pyrolysis temperatures typically range between 300 and 900 °C [12,14,16,32,44,45,46,47,48], with optimal biochar properties generally recorded at temperatures between 500 and 750 °C [14,44,49,50,51]. At these temperatures, the carbonization process enhances the stability and quality of the resulting biochar, improving its adsorption capabilities and nutrient retention [49]. The pyrolysis yields three primary products: gases (including carbon dioxide, carbon monoxide, and hydrocarbons), liquids (such as organic solutions and tar), and solids (char) [52]. The specific proportions and compositions of these products vary based on the type of biomass used, the pyrolysis method, and the reactor design employed [53].

Various types of reactors can be utilized for the pyrolysis of SMS, including batch, continuous, and fluidized bed reactors. Batch reactors are straightforward and allow for flexibility in feedstock processing but may have lower efficiency. Continuous reactors, on the other hand, facilitate a more efficient and scalable process by continuously feeding SMS into the reactor, thereby enhancing productivity. Fluidized bed reactors are particularly advantageous as they provide excellent heat transfer and uniform temperature distribution, which are critical for consistent biochar quality [54]. By carefully selecting the pyrolysis conditions and reactor type, producers can optimize the characteristics of SMS-derived biochar for various applications.

### 3.2. Influence of Parameters on Biochar Properties

The production of SMS-derived biochar is significantly influenced by various pyrolysis parameters, including the conditions used in pyrolysis (temperature, pressure, heating rate, final temperature, residence time, reactor atmosphere, and feed system), the type of reactor (which affects the heating rate and process duration), and the characteristics of the feedstock (particle size, type, and composition) [51,53,55]. Higher pyrolysis temperatures typically enhance the carbon content, surface area, pore size, and some nutrients, like the P and K contents of the resulting biochar, promoting a more stable structure [16,51]. For example, biochar produced at temperatures exceeding 500 °C typically exhibits high hydrophobicity, increased surface area, and higher micropore volume, making it suitable for adsorbing organic pollutants. In contrast, biochar produced at lower temperatures (below 500 °C) retains more oxygenated functional groups, which are advantageous for immobilizing inorganic pollutants [56,57]. These characteristics highlight the importance of optimizing pyrolysis conditions to tailor SMS-derived biochar to specific environmental and agricultural applications.

Residence time during pyrolysis plays a vital role in determining the quality and properties of SMS-derived biochar. Extended residence times can enhance carbonization but may also lead to the loss of volatile compounds, reducing nutrient availability, and potentially degrading overall biochar quality [10,58]. Additionally, variations in the feedstock type, such as different mushroom species or substrates, can affect the physicochemical properties of the biochar, including its pH, CEC, essential nutrients, and overall stability [7,10]. Understanding these mechanisms is essential for optimizing biochar production processes to achieve specific characteristics suited for various applications, from soil enhancement to environmental remediation [59]. Overall, careful control of pyrolysis conditions can produce biochar from SMS with desirable properties, while preparing the raw material by reducing its moisture content to below 15% before firing ensures higher biochar yield and quality, extends furnace lifespan, and minimizes emissions. This enhances efficiency in agricultural and ecological applications as the carbon in biochar also contributes to reducing greenhouse gases [12,60].

Furthermore, the effectiveness of SMS-derived biochar can be enhanced through pre- and post-treatment methods that modify its surface chemistry, porosity, and adsorption capacity for targeted applications [61]. Pre-treatment often involves impregnating biochar feedstocks with metallic (e.g., aluminum, iron, manganese, and zinc) or non-metallic (e.g., nitrogen, sulfur, phosphorous, and boron) elements before pyrolysis, creating engineered carbon structures that boost adsorption efficiency [62,63]. However, concerns over metal leaching have led to interest in co-doping strategies, where metal/non-metal combinations enhance stability [61,64]. Post-treatment methods, such as acid or alkaline washing, alter surface functional groups and increase surface area [65]. Additionally, steam modification and gas purging (CO_2_ and NH_3_) further optimize micropore structures and chemical properties [66]. These modifications allow for biochar to be tailored to specific contaminants, making it a highly adaptable material for environmental remediation and soil enhancement in agricultural systems.

### 3.3. Energy Efficiency and Economic Viability

Producing biochar from SMS presents an opportunity for energy efficiency (biochar, bio-oil, and bio-gas) while contributing to the commercial, industrial, and economic benefits of society [9,27,67]. The carbonization process, which involves pyrolyzing SMS at high temperatures, not only transforms this agricultural waste into a valuable resource but also captures the energy released during the process. This captured energy can be utilized to power biochar production itself, thereby improving the overall energy efficiency of the operation [13,68,69]. Studies indicate that SMS-derived biochar has an energy content ranging from 17.32 to 22.72 MJ/kg, highlighting its potential as a renewable energy source [32,44,70,71].

Utilizing inexpensive lignocellulosic residues offers a cost-effective and environmentally friendly approach to substrate production, facilitating their efficient management and valorization [6]. Economically, producing biochar from SMS is notably more affordable ($60–117.50/ton or $0.06–0.1175/kg) compared to conventional feedstocks such as oak wood ($0.77), coconut shell ($0.80), *Oiltea camellia* shell ($0.67), chicken manure ($1.30), and virgin wood feedstock ($17.8) [14,50,72,73]. The use of widely available agricultural byproducts significantly lowers raw material costs, benefiting smallholder farmers and increasing the profitability of mushroom producers [14]. Cost analyses reveal that SMS-derived biochar production can be competitive, with production costs varying based on technology and production scale [14,72,73]. Moreover, the potential for additional revenue streams through the sale of biochar as a soil amendment or as a carbon offset can further enhance its economic viability. The combination of energy recovery, reduced production costs, and the generation of valuable byproducts positions SMS-derived biochar as a sustainable and economically attractive alternative in the agricultural and energy sectors [8,74]. As research continues to optimize environmentally friendly production methods and develop processes that reduce carbon dioxide emissions into the environment, such as utilizing waste heat to pre-dry high-moisture raw materials before pyrolysis, the anticipated economic benefits are expected to increase, making SMS-derived biochar a promising solution for sustainable agricultural practices and waste management.

## 4. Physicochemical and Biological Properties of SMS-Derived Biochar

### 4.1. Structural, Physical, and Chemical Properties

The physicochemical properties of SMS-derived biochar are pivotal in determining its effectiveness as both a soil amendment and environmental remediation tool [7]. The characteristics of biochar vary depending on the mushroom species from which the SMS is derived (Table 2), but some common features stand out. One key attribute is its pore structure, which significantly impacts its specific surface area (SSA) and particle size (PS) [7,14]. SMS-derived biochar often exhibits a substantial SSA, ranging from 3.09 to 1095 m^2^g^−1^, enabling it to efficiently adsorb nutrients and contaminants [7,16,20,48]. Its PS, typically between 0.92 and 50 mm, enhances interactions with soil components while promoting root penetration and aeration in plants [16,20,33,46,48].

Chemically, SMS-derived biochar features a pH range of 6.49 to 12.31, making it suitable for neutralizing acidic soils. Its EC, measured at 2.09 to 3.54 dS m^−1^, indicates efficient ionic mobility in the soil. Nutritionally, it is enriched with macronutrients such as N (0.44–2.71%), P (0.03–3.80%), and K (0.08–1.39%), along with significant micronutrients, including Ca (0.40–9.69%), Mg (0.50–1.70%), and trace elements like Fe (1500–24,000 mg·kg^−1^), Mn (10–1200 mg·kg^−1^), Zn (84–1000 mg·kg^−1^), Cu (10 mg·kg^−1^), and Na (290–1100 mg·kg^−1^). This nutrient wealth supports plant growth and fosters beneficial microbial activity in the soil.

Another standout property is its CEC, which measures its ability to retain and release nutrients to plants. With CEC values between 19.37 and 32.29 cmol kg^−1^, SMS-derived biochar serves as an effective nutrient reservoir [7]. Additionally, its high carbon content ensures exceptional stability in the soil, resisting microbial degradation and maintaining its benefits for a long time [7,14]. Interestingly, the stability and high carbon content of SMS-derived biochar make it suitable for reuse in mushroom cultivation. Its strong carbon–carbon double bonds resist reactions with water or lime during the preparation of mushroom substrates at room temperature [14,20,75]. This durability and versatility position SMS-derived biochar as an ideal solution for sustainable agriculture and environmental management, offering long-term benefits for soil health, productivity, and ecosystem resilience.

### 4.2. Capability of Heavy Metal Adsorption

Biochar derived from SMS has shown significant potential in addressing heavy metal contamination in soils and water sources, offering a sustainable and effective solution for soil remediation and environmental protection [7,16,49]. Several studies have demonstrated that biochar, regardless of its source, can reduce the bioavailability of heavy metals in contaminated soils and polluted wastewater, thus limiting their potential to enter the food chain and reducing ecological risks [7,13,15,16,76]. Specifically, SMS-derived biochar has been recognized for its ability to adsorb and immobilize heavy metals such as cadmium (Cd), chromium (Cr), Cu, lead (Pb), and Zn, making it an excellent option for treating contaminated water sources, as well as for protecting human health from long-term exposure to these toxic elements [15,41,77].

The unique physicochemical properties of SMS-derived biochar, including its high surface area, porous structure, and active functional groups, contribute to its exceptional ability to bind heavy metals [49]. These characteristics enable SMS-derived biochar to reduce the mobility and bioavailability of these metals in the soil, which prevents their uptake by plants and reduces the associated environmental risks [15,41,77]. Furthermore, the stable carbon matrix and high carbon content of SMS-derived biochar enhance its long-term effectiveness by preventing the leaching of metals into groundwater, offering a lasting solution to soil contamination [59,78]. The nutrient-rich composition of SMS-derived biochar also supports the growth of beneficial microorganisms [7], which can further aid in the degradation or transformation of pollutants, leading to a healthier and safer soil environment [31,55].

The adsorption capabilities of biochar produced from SMS for the removal of heavy metals are comprehensively described in Table 3, demonstrating its potential as an economically feasible and environmentally conscious remediation method. The data show a considerable variation in adsorption efficiency depending on the type of mushroom substrate used and the specific heavy metal being targeted.

Among the various SMS-derived biochars, *A. bisporus*-derived biochar exhibited strong adsorption capacities for cadmium (64.8 mg g^−1^), copper (68.1 mg g^−1^), and zinc (55.2 mg g^−1^), making it a viable option for treating cadmium- and copper-contaminated environments. Similarly, *A. auricula*-derived biochar demonstrated the highest chromium (118.0 mg g^−1^) and cadmium (114.6 mg g^−1^) removal efficiencies, indicating its potential application in chromium-contaminated sites. The biochar produced from *L. edodes* and *P. ostreatus* showed remarkable adsorption capacities for lead, with values reaching 398 mg g^−1^ and 326 mg g^−1^, respectively. Notably, *G. lucidum*-derived biochar exhibited a wide range of cadmium (23.81–75.82 mg g^−1^) and lead (141.59–262.76 mg g^−1^) adsorption efficiencies, suggesting that its adsorption performance may be influenced by processing conditions or biochar modification techniques. In addition, spent mushroom compost-derived biochar demonstrated exceptionally high adsorption capacities across multiple heavy metals, including copper (52.6–364 mg g^−1^), lead (564 mg g^−1^), and zinc (332 mg g^−1^). These results underscore its broad-spectrum potential for wastewater treatment and heavy metal detoxification.

Overall, the findings demonstrate that SMS-derived biochar has an interesting potential as an efficient heavy-metal-removal adsorbent, with some mushroom substrates showing greater attraction for various metal ions. Further research into improving the conditions and modification methods used to produce biochar may increase its adsorption efficiency, providing the possibility for scalable uses in environmental remediation and sustainable crop waste management.

### 4.3. Long-Term Stability and Environmental Persistence

The stability and environmental persistence of SMS-derived biochar are essential for its effectiveness as a soil amendment [59]. Research studies indicate that SMS-derived biochar is highly stable, owing to its unique physicochemical properties, such as an elevated carbon content and a porous structure. These features enhance its resistance to microbial degradation, enabling it to remain in the soil for prolonged periods while gradually enhancing soil health and fertility [14,55,59]. The slow decomposition of biochar not only aids in carbon sequestration but also minimizes nutrient leaching, thereby improving nutrient availability for plants over time [34]. Additionally, its incorporation into mushroom-growing substrates also boosts yields, retains substrate moisture, and minimizes nutrient loss [14]. One might assume that this helps maintain harvest stability and yield quality consistency. Consequently, integrating SMS-derived biochar into agricultural practices offers a sustainable approach to improving soil quality while contributing to long-term environmental objectives.

### 4.4. Beneficial Microbial Communities

SMS-derived biochar promotes the growth of beneficial microbial communities in the soil, which in turn supports enhanced plant health and growth. Its porous structure and nutrient-rich composition create an ideal habitat for microorganisms, particularly nitrogen-fixing bacteria such as plant growth-promoting rhizobacteria (PGPR), which play a key role in nutrient treatment and plant health [16]. The porous nature of biochar, consisting of micropores (less than 2 nm), mesopores (2–50 nm), and macropores (more than 50 nm), offers an ideal habitat for a variety of microorganisms, such as bacteria, protozoa, and fungi [82]. These microhabitats support microbes from leaching, competition, predators, toxins, and moisture loss, fostering their activity and interaction [83]. The macropores of biochar are well-suited for microbial colonization, while mesopores and micropores store dissolved nutrients and water, supplying the resources needed for microbial metabolism [84,85]. This synergy contributes to improved plant growth and resilience, aligning with recent advancements in microbe-assisted plant development strategies [86]. Moreover, when used in combination with microorganisms, SMS-derived biochar can improve soil structure and increase nutrient cycling, thereby boosting nutrient availability and plant productivity [16,87]. Studies also suggest that combining biochar with mycorrhizal fungi can enhance soil structure, disease resistance, and plant growth [88,89,90], opening up new avenues for further research on SMS-derived biochar. This dynamic relationship between biochar and microbes supports sustainable agricultural practices, contributing to long-term improvements in soil health and ecosystem balance.

In wastewater treatment, biochar also provides a stable environment for pollutant-degrading bacteria, such as *Acinetobacter* [91], *Pseudomonas putida* [92], and *Rhodobacter capsulatus* [93], which help break down organic contaminants and facilitate nitrogen removal through nitrification and denitrification [94]. Combining SMS-derived biochar with various types of microbial life might extend the possibility of sustainable wastewater management by greatly enhancing the removal of heavy metals from wastewater, especially in the agricultural sector.

## 5. Applications of SMS-Derived Biochar in Modern Agricultural Systems

**Figure 1:**
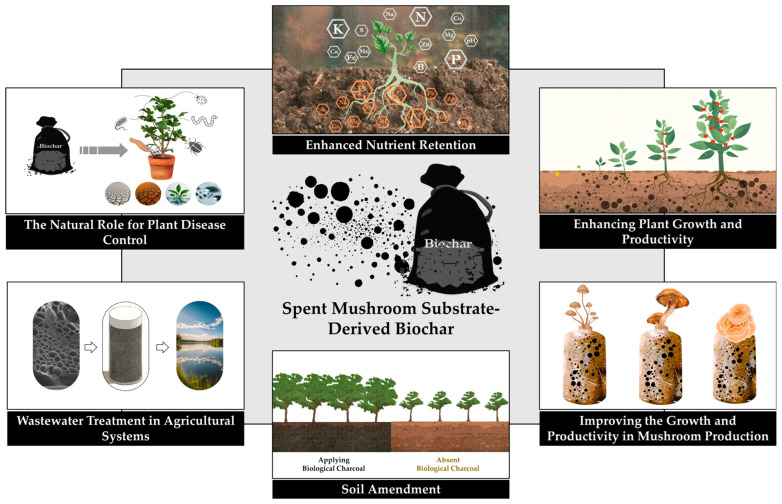
Applications of biochar produced from SMS in modern agricultural practices.

The application of SMS-derived biochar in modern agricultural systems offers numerous benefits (Figure 1), including enhanced soil amendment, improved nutrient retention, and mechanisms that promote plant growth and productivity [8,16,30,35]. Its utility extends to wastewater treatment in agricultural settings, where it aids in contaminant removal [21], and it plays a natural role in plant disease control by fostering effective microbial ecosystems [19]. Additionally, SMS-derived biochar significantly improves growth and productivity in mushroom production [14], making it a versatile tool for sustainable agriculture.

### 5.1. Soil Amendment

SMS-derived biochar plays a crucial role as a soil amendment in modern agricultural systems, particularly in enhancing soil fertility, improving water retention, and increasing organic matter content [7,8,35]. When incorporated into the soil, biochar improves the structure, porosity, and overall physicochemical and biological properties of the soil, facilitating better aeration and root penetration. Its high surface area and porous characteristics enable greater water retention, allowing soils to hold moisture for extended periods [7,95]. This capability mitigates irrigation needs and enhances drought resilience, which is particularly valuable in regions facing water scarcity [33].

SMS-derived biochar not only enhances soil physical properties but also plays a crucial role in reducing nutrient leaching while enriching the soil with essential nutrients and organic matter. Its composition includes macronutrients like N, P, and K, which directly support plant growth and improve crop yields, alongside micronutrients such as Ca [13,16]. Research by Lou et al. [35] highlighted that SMS-derived biochar exhibits excellent nutrient retention efficiency, as confirmed by leaching tests. The organic matter in this biochar encourages beneficial microbial activity, which is fundamental to enhancing soil fertility and nutrient cycling [96]. Furthermore, its application has been shown to improve the CEC of soils, increasing their ability to retain and make essential nutrients available to plants. This property is critical for maintaining long-term soil fertility and advancing sustainable agricultural practices [7]. Incorporating SMS-derived biochar as a soil amendment is a promising strategy for improving soil health and productivity. It offers an effective means of conserving nutrients without the need for additional materials, contributing significantly to sustainable agricultural systems and long-term ecological balance [35].

### 5.2. Enhanced Nutrient Retention

SMS-derived biochar significantly enhances nutrient retention in soils, providing a mechanism that improves the availability of essential nutrients to plants [7]. The porous structure of biochar increases the surface area, allowing it to adsorb and retain essential macronutrients and micronutrients. This adsorption occurs through various mechanisms, including electrostatic attraction, cation exchange, and the formation of surface complexes with nutrient ions. Moreover, the high CEC of SMS-derived biochar enables it to hold onto positively charged nutrient ions (cations) and release them slowly over time [82]. This slow-release characteristic is beneficial as it helps maintain a steady supply of nutrients in the soil, reducing the risk of nutrient leaching into water systems and promoting more efficient use of fertilizers, thus serving as a sustainable alternative to expensive chemical fertilizers [97,98]. Additionally, the organic matter in biochar enhances microbial activity, which further aids in the breakdown of organic compounds and the mineralization of nutrients, making them more accessible to plants. This combination of enhanced nutrient retention and gradual-release mechanisms not only improves soil fertility but also contributes to the sustainability of agricultural systems by reducing the need for synthetic fertilizers and minimizing environmental impacts associated with nutrient runoff [99]. Therefore, the incorporation of SMS-derived biochar into soil management practices represents a promising strategy for optimizing nutrient availability and promoting healthier and more productive crops [7,16].

### 5.3. Enhancing Plant Growth and Productivity

Applying biochar has generally been demonstrated to significantly improve plant growth and yield through several mechanisms, primarily involving nutrient retention, microbial activity, soil structure enhancement, and increased efficiency in plant nutrient uptake [100], making them available to plants over an extended period. This nutrient retention capacity reduces leaching and improves the efficiency of fertilizer use, contributing to higher crop yields. Moreover, the addition of biochar promotes beneficial microbial activity in the soil [100,101]. The organic matter present in SMS-derived biochar provides a habitat and food source for a diverse range of soil microorganisms. These microbes play a crucial role in nutrient cycling, breaking down organic materials, and enhancing nutrient availability to plants [99]. Several studies have shown that crops such as cauliflower, lettuce, and rapeseed exhibit increased growth and yield when planted in soil treated with SMS-derived biochar, demonstrating the positive impact of biochar on crop productivity [15,50,87]. In practical applications, experiments with various crops have yielded promising results. For example, research has indicated that incorporating SMS-derived biochar into the soil improved the growth of lettuce and other leafy vegetables by enhancing nutrient uptake and promoting healthier root systems [16,87,102]. In another study, experiments using *Pleurotus* SMS and its biochar in tissue culture of *Clinacanthus nutans* revealed promising outcomes, such as increased leaf numbers and shoot length, although the effects varied with concentration. While SMS supports plant development, SMS-derived biochar shows potential as a nutrient adsorbent. These applications highlight biochar’s role in advancing green biotechnology and zero-waste initiatives by transforming agricultural waste into valuable resources [103] and supporting sustainable farming practices.

### 5.4. Wastewater Treatment in Agricultural and Environmental Systems

SMS-derived biochar has shown great promise in wastewater treatment applications due to its excellent adsorption properties, making it effective for removing contaminants from industrial sectors to agricultural runoff [15]. The porous structure and high surface area of biochar allow it to adsorb a range of pollutants, including heavy metals and pesticide residues, which are common in agricultural wastewater [16,100]. Studies have demonstrated that SMS-derived biochar can significantly reduce concentrations of heavy metals like Pb, Cd, Cr, Cu, and Zn, effectively improving water quality and minimizing environmental impact [15,30,47,77,80,81,104]. Additionally, SMS-derived biochar enhances the immobilization of heavy metals and organic contaminants like pesticide residues through adsorption [7]. For example, recent research demonstrated its effectiveness in reducing pyrethroid pesticide levels, such as tetramethrin, beta-cypermethrin, and fenvalerate, in water samples (tap water, lake water, and river water), contributing to safer crop management and sustainability [105]. This simple and efficient method has practical potential for detecting and managing contaminants in various water sources. Beyond water treatment, the integration of SMS-derived biochar into agricultural practices supports a circular economy by converting agricultural byproducts into valuable resources [6,21]. These applications highlight the potential of SMS-derived biochar as a sustainable solution for improving water quality in agricultural landscapes while promoting environmental health.

### 5.5. Natural Role for Plant Disease Control

The application of SMS-derived biochar demonstrates a high positive correlation with plant growth, yield, and biochemical responses, making it a valuable tool in sustainable agriculture [16]. One of its most notable benefits is its dual ability to manage plant pathogens while enhancing soil health. By improving the soil’s physical and chemical properties and fostering beneficial microorganisms, biochar supports plant defense mechanisms against diseases [19]. For example, biochars derived from citrus wood and greenhouse wastes have been shown to induce systemic resistance against *Botrytis cinerea* in various hosts, including *Lycopersicon esculentum* (tomato), *Capsicum annuum* L. cv. Maccabi (bell pepper), and *Fragaria ananassa* (strawberry). Similarly, citrus and pine wood biochars have been effective in resisting diseases caused by *Colletotrichum acutatum* in strawberries, *Leveillula taurica* in bell peppers, and *Phytophthora cactorum* in red maple trees [17,19,106]. Numerous studies have demonstrated that biochar applications can lead to healthier crops by suppressing soil-borne pests and pathogens [17,19,107,108]. Its porous structure provides habitats for beneficial microorganisms, such as rhizobia, mycorrhizal fungi, actinomycetes, diazotrophic bacteria (plant-growth-promoting bacteria (PGPB)), and other soil microbiomes, which form symbiotic relationships with plant roots. These microbes contribute to nutrient mineralization, hormone production, and competitive inhibition of harmful pathogens, enhancing overall plant resilience [17,109]. Moreover, biochar’s ability to boost microbial biomass and alter microbial community structures further supports diverse microbial activity by modifying soil physicochemical properties, ultimately benefiting plant growth and sustainability [110,111]. Research also indicates that using biochar at concentrations of 1% and 2% can significantly reduce the incidence of diseases in tomatoes, such as *Fusarium* wilt, thereby enhancing plant growth and productivity [112]. Additionally, biochar inhibits plant pathogenicity by enhancing resistance, increasing nutrient availability, and detoxifying pollutants, further reducing the stressful environment for plants and enhancing resistance to pests [113]. These benefits suggest the possibility that SMS-derived biochar not only improves soil fertility and structure but also offers a strategic eco-friendly solution for managing soil-borne pests and diseases. By integrating biochar into agricultural systems, farmers can enhance productivity while promoting sustainability.

### 5.6. Improving the Growth and Productivity in Mushroom Production

The incorporation of SMS-derived biochar into mushroom production substrates has demonstrated significant potential in enhancing the mycelial growth and yield of various mushroom species, especially oyster mushrooms like *P. ostreatus* and *P. pulmonarius* [14,20,36,114]. When added to growing media, biochar improves the substrate’s aeration and water-holding capacity, creating a more conducive environment for mycelium development. This increased porosity facilitates better oxygen flow and moisture retention, both of which are critical for optimal mycelial growth [14,20]. As a result, mushroom farmers may have the chance to obtain higher-quality fruiting bodies and increased yields compared to traditional substrates that do not contain biochar. Moreover, SMS-derived biochar is rich in essential nutrients, trace elements, and carbon, which enrich the growing medium and can help the mycelium grow more effectively and provide the mushrooms with a consistent supply of nutrients [20].

Numerous studies have shown that incorporating SMS-derived biochar into the substrate enhances nutrient availability, leading to faster colonization and increased fruiting. For instance, research by Hu et al. [14] indicated that using SMS-derived biochar resulted in 20 to 25% higher yields and reduced fruiting times by 4 to 6 days compared to conventional SMS. Similarly, other studies have reported that adding biochar to the growing substrate for oyster and button mushrooms leads to increased productivity and extended harvesting cycles [14,20,114,115,116,117]. Recent research, such as that by Kansara et al. [118], explored SMS-derived biochar produced from species like shiitake (*L. edodes*), blue oyster (*P. ostreatus*), and lion’s mane (*H. erinaceus*). Trials with biochar at different mass loadings revealed notable improvements, particularly for lion’s mane, with biochar concentrations around 5% significantly boosting yields. The findings highlight reduced colonization times and increased fruit body production, demonstrating biochar’s role in enhancing substrate efficiency. Additionally, biochar stability slows substrate degradation and minimizes nutrient loss, ensuring sustained nutrient availability and promoting long-term cultivation efficiency [14,20]. These benefits, combined with the environmental advantages of waste heat recovery and circular waste-to-biochar processes, make SMS-derived biochar a sustainable choice for mushroom farming. Its adoption supports both commercial and small-scale growers, aligning with environmentally friendly cultivation practices in contemporary agricultural systems.

## 6. Comparison of SMS-Derived Biochar to Other Biochars

### 6.1. Performance Comparison of SMS-Derived Biochar to Other Biochars

The comparison between SMS-derived biochar and other biochar highlights the distinctive properties and advantages of SMS-derived biochar (Table 4). These unique characteristics make it a superior option in various agricultural and environmental applications. In terms of superior chemical and physical properties, SMS-derived biochar boasts a wide pH range (6.5–12.3), catering to diverse soil needs, whereas many other biochars, like those from acacia or bamboo wood, exhibit narrower pH ranges (e.g., 5.2–8.83). The C/N ratio of SMS-derived biochar (26.2–85.2) demonstrates balanced carbon and nitrogen content, promoting sustained microbial activity and nutrient cycling. In contrast, conventional options such as bagasse (C/N ~118) may limit microbial functionality due to excessive carbon.

The organic carbon content of SMS-derived biochar (37.5–70.7%) aligns with or surpasses that of traditional biochars, contributing to long-term soil carbon sequestration. Additionally, it boasts a higher nutrient density, with N (0.3–7.2%), P (0.03–3.8%), and K (0.08–1.4%) present in greater diversity and quantity than in biochars such as corn cob (1.2–4.3% N, 0.31% P, and 1.8% K), acacia trees (0.6–1% N, 0.1–1.14% P, and 0.71% K), or bagasse (0.6% N, 0.08% P, and 0.43% K). These macronutrients, paired with substantial micronutrient levels (e.g., up to 13.4% Ca, 0.5–1.7% Mg, and 0.04–1.6% S), create a superior amendment for enhancing soil fertility, often absent in some biochars from traditional feedstocks. Notably, its metal content, including iron (1500–24,000 mg·kg^−1^), manganese (10–1200 mg·kg^−1^), and zinc (84–1000 mg·kg^−1^), facilitates enhanced plant nutrition. These values significantly outpace those of other biochars, such as bamboo wood or rice husk, which have far lower micronutrient concentrations.

In terms of surface area and porosity, SMS-derived biochar exhibits superior surface area (SSA: 3.1–1095 m^2^ g^−1^) and porosity, enabling improved water retention, aeration, and pollutant adsorption. This surpasses many other biochars, like coconut shell (SSA: ~613–626.8 m^2^ g^−1^) or corn cob (SSA: ~53.7–56.9 m^2^ g^−1^), which have limited adsorption capacities. The pore size distribution of SMS-derived biochar (0.9–50 nm) optimally supports microbial colonization, enhancing soil health and plant growth.

SMS-derived biochar also excels in versatility and environmental remediation. Its ability to adsorb heavy metals and pollutants surpasses that of traditional biochars due to its higher nutrient density and structural properties [100]. Moreover, studies demonstrate its exceptional performance in promoting mycelial growth and improving mushroom yields when incorporated into cultivation substrates [14,20,36,114], a feature unmatched by other biochars. While other biochars have merits, SMS-derived biochar outperforms in chemical richness, structural attributes, and versatility.

Another crucial aspect of its enhanced performance is the presence of nitrogen (N)-doped biochar, which has received significant attention due to its ability to modify surface morphology and properties after high-temperature pyrolysis [123]. These N-functionalized biochars improve cation exchange capacity, surface reactivity, and adsorption efficiency, making them essential for soil fertility enhancement, pollutant removal, and microbial interactions [124,125]. Notably, SMS from shiitake mushroom cultivation has been used as a carbon precursor to produce nitrogen-doped activated biochar, which has demonstrated efficiency in removing reactive orange-16 azo dye from water and treating contaminants in synthetic effluents and real sewage water. This highlights SMS as a valuable waste resource for activated carbon production, with N-doping significantly boosting adsorption performance [126]. However, advanced analytical techniques like Raman spectroscopy, X-ray photoelectron spectroscopy (XPS), nuclear magnetic resonance (NMR), thermogravimetric analysis (TGA), Brunauer–Emmett–Teller (BET) analysis, Fourier-transform infrared spectroscopy (FTIR), and scanning electron microscopy (SEM), among others, are crucial for identifying important N-functional groups (such as pyridinic-N, pyrrolic-N, graphitic-N, and oxidized-N) for the purpose of contributing to the characterization of these features. These functional groups play a critical role in nutrient retention, heavy metal adsorption, and catalytic activity [126,127]. With many applications in environmental management and sustainable agriculture, SMS-derived biochar is positioned as a high-end option for contemporary methods that promote ecological sustainability and productivity.

### 6.2. Economic Costs, Impact Analysis, and Environmental Effects

A comparison of the economic costs and environmental impacts of SMS-derived biochar and traditional biochars highlights significant advantages of SMS-derived biochar in terms of cost-effectiveness and sustainability (Table 5). SMS-derived biochar, offers a cost-effective alternative to biochars produced from conventional biomass feedstocks such as hardwoods, agricultural residues, and tree branches. This is largely due to the abundance and low cost of SMS, a byproduct of mushroom cultivation, which eliminates the need for expensive raw materials. For instance, SMS-derived biochar costs approximately $117.5/ton in Chongqing, China [14], significantly lower than traditional biochars, such as those derived from bamboo ($7000 in Alabama, USA) or switchgrass ($5490 in the USA). The economic disparity becomes even more apparent when comparing SMS-derived biochar to biochars made from virgin wood feedstocks ($17,800 in Massachusetts, USA) or tree branches ($11,000 in Kansas, USA). Even moderately priced biochars, such as those from coconut shells ($800 in the USA) or sewage sludge ($560–1000 in the USA) [128,129], do not match the affordability of SMS-derived biochar. This cost advantage makes SMS-derived biochar an attractive option for large-scale applications, particularly in agriculture and environmental management.

The processing of SMS into organic fertilizers represents a common method of waste management, with value for its potential economic benefits [121]. A critical metric for assessing the practical value of recycled SMS is its input–output ratio compared to that of traditional organic fertilizers. According to Hu et al. [14], the production costs of SMS-derived biochar and organic fertilizers encompass four main components, i.e., facilities, energy, transportation, and labor, while the outputs are measured in terms of increased crop yields and cost savings. For a production volume of 1000 kg, the economic analysis reveals that the production cost of SMS-derived biochar is approximately $117.50, with potential economic benefits of around $414.80. In contrast, the cost of organic fertilizers is about $63.90, yielding economic benefits of $193.30. Although SMS costs are roughly double that of organic fertilizers, the output-to-input ratio for SMS-derived biochar stands at 4.53, compared to 4.02 for organic fertilizers, resulting in a total benefit for SMS-derived biochar that exceeds organic fertilizers by $221.50.

According to a case study by Zhang et al. [130], China generates around 15 million tons of SMS annually from edible mushroom production. If SMS-derived biochar manufacturing techniques were adopted nationally, the value added could reach an impressive $3.32 billion beyond that of organic fertilizers. This increase in productivity is associated with the reduced consumption of ecological resources and lower carbon emissions per agricultural product unit. Additionally, the conversion of SMS to SMS-derived biochar does not require fermentation, minimizing environmental risks and saving both time and space [14]. This process aligns with the principles of clean production, making it a more sustainable option than traditional organic fertilizers.

When examining the budget of biochar production enterprises, the baseline costs for producing 1 Mg (1000 kg) of biochar are approximately $717.76, excluding uncertainties in various parameters. The economic impacts of biochar production suggest an output-to-input ratio ranging from 2.01 to 2.85 (mean: 2.43) [131]. Notably, SMS-derived biochar production boasts variable costs that are six times lower than those of other biochar sources and offers a higher output-to-input ratio of 1.86 times. These findings highlight the economic viability and environmental benefits of utilizing SMS as a feedstock for SMS-derived biochar, promoting sustainable agricultural practices while effectively managing waste and reducing ecological impacts.

Beyond cost, SMS-derived biochar also excels in its environmental benefits. Utilizing SMS for biochar production addresses the waste management challenges associated with spent substrate disposal, effectively reducing the environmental footprint of mushroom farming. This conversion process recycles SMS into a valuable product that enhances soil health, improves nutrient retention, and supports pollution remediation [7,15]. Additionally, the multifunctionality of SMS-derived biochar further strengthens its environmental appeal. Its uses in agriculture, like improving soil fertility and supporting environmentally friendly farming practices, help ecological sustainability in the long term [7]. All things considered, SMS-derived biochar is notable for its minimal cost of production, effective waste management, and positive environmental impacts. By addressing both economic and ecological concerns, SMS-derived biochar presents itself as a superior alternative to traditional biochar, aligning with the goals of sustainable agriculture and innovative environmental practices.

## 7. Biochar Regeneration and Its Possibilities for Long-Term Sustainability

Biochar is widely regarded as being cost-effective because of its simple production processes and inexpensive feedstock. One significant advantage is its regenerability, which significantly reduces operational costs and allows for multiple reuse cycles in wastewater management [132]. Chemical regeneration has been utilized frequently to preserve the adsorption capability of biochar, providing a long-term sustainable solution. These techniques involve action treatment with salts, metal oxides, acids, and alkalis to enhance biochar’s effectiveness [133]. However, challenges such as secondary contamination and varying regeneration efficiency, depending on adsorption mechanisms and pore structure, must be addressed. Various regeneration techniques have been developed, which are categorized into desorption (including thermal methods, such as steam, inert gas, hot water, and microwave, as well as non-thermal methods like chemical-based substances, surfactants, and supercritical fluids) and decomposition (including oxidation, electrochemical treatment, ultrasound, and microbiological processes) [132]. While thermal methods effectively break down pollutants, they are energy-intensive and costly. In contrast, non-thermal techniques, particularly chemical regeneration, offer a more sustainable and cost-efficient approach by preserving biochar’s structure for repeated use [134,135]. This report demonstrates the necessity of more research to optimize regeneration effectiveness and support the use of biochar in sustainable wastewater management.

## 8. Potential Benefits Beyond Agriculture

**Figure 2:**
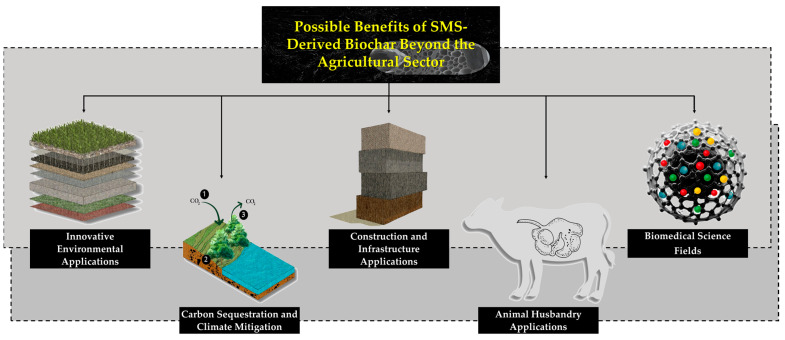
Potential advantages of biochar derived from SMS beyond agriculture.

Biochar offers numerous potential benefits beyond agriculture, including its use in animal husbandry for improving livestock health and reducing odors, enhancing carbon sequestration to combat climate change, and innovative applications in environmental management, such as wastewater treatment and pollution mitigation. Furthermore, its structural properties lend themselves well to construction and infrastructure projects, making it a versatile material in sustainable development initiatives (Figure 2) [95]. These diverse applications demonstrate the flexibility of biochar and create opportunities for SMS-derived biochar to be utilized beyond agricultural fields, extending its impact across multiple sectors.

### 8.1. Innovative Environmental Applications

A carbon-rich product produced by pyrolyzing organic materials, biochar has been proposed as the perfect substrate additive and offers creative uses in urban infrastructure to enhance the quality of runoff from green roofs. Its effective nutrient sorption capacity and ability to promote plant growth make it a perfect choice for urban gardening projects. By enhancing soil aeration and nutrient availability, biochar fosters healthier plant ecosystems in urban environments [136,137]. Moreover, incorporating biochar into city planning not only supports sustainable urban agriculture but also contributes to climate resilience, aligning with broader environmental management goals [138]. Research indicates that biochar derived from various sources, such as hardwoods, eucalyptus, mixed conifer wood, green waste, sewage sludge, coconut shell, and waste wood, can enhance both aesthetic and ecological outcomes in urban settings [136,139,140,141,142,143,144]. This presents another strategy for the potential of SMS-derived biochar as a sustainable urban infrastructure innovation, especially for green roofs.

### 8.2. Carbon Sequestration and Climate Mitigation

SMS-derived biochar plays a crucial role in carbon sequestration and climate mitigation by capturing atmospheric carbon and reducing overall greenhouse gas emissions [7,8,12]. When applied to soils, biochar enhances carbon storage and delivers numerous productivity benefits, including reduced bulk density, improved water retention, increased nutrient availability, stabilization of organic matter, and enhanced microbial activity, along with the ability to sequester heavy metals. Its stable carbon structure inhibits the rapid breakdown of organic matter, a primary contributor to carbon release, while also increasing phosphorus availability in highly weathered soils [11,100,145]. Converting local feedstocks and agricultural waste to biochar is particularly beneficial for smallholder farming, tree nurseries, and specialty crop management as it promotes soil health and fertility, leading to greater plant growth and further carbon absorption [145]. Numerous studies indicate that incorporating biochar into agricultural practices significantly reduces carbon emissions, positioning it as an effective strategy for climate change mitigation [7,10,146]. Utilizing SMS for biochar production not only supports agricultural sustainability but also contributes to global climate action efforts.

### 8.3. Construction and Infrastructure Applications

Incorporating biochar into construction materials is increasingly recognized for its potential to enhance building performance and sustainability [137]. Numerous studies show that adding biochar to building materials, such as bricks, concrete, and cement, can improve thermal insulation, reduce overall weight, and boost energy efficiency in buildings. This eco-friendly approach not only repurposes agricultural byproducts but also aligns with the construction industry’s transition toward sustainable practices [137,147,148]. This approach suggests the integration of SMS-derived biochar in building practices, leading to the creation of more environmentally friendly structures that also advance innovative material science.

### 8.4. Animal Husbandry Applications

Several studies have shown that biochar supplementation in animal husbandry, like cows, pigs, poultry, and fish, can significantly enhance animal growth, improve hematological profiles, and boost the yield of meat, milk, and eggs [95,149,150]. Additionally, biochar has been shown to reduce methane emissions in ruminants, a crucial benefit for minimizing livestock’s environmental impact. When mixed into animal diets, biochar supports nutrient absorption, aids in digestion, and removes contamination, all of which help the overall health and growth of the animal. Additionally, biochar use in livestock bedding or manure management reduces odors and pathogen presence, fostering a healthier environment [149]. Crucially, biochar in animal systems also helps in the recovery of essential nutrients from waste and raises the value of fertilizer made from manure, which may subsequently be recycled into agricultural soils, reducing negative impacts on the environment and promoting sustainable nutrient cycles [151]. This strategy demonstrates the additional benefits that SMS-derived biochar might offer for future sustainable animal husbandry.

### 8.5. Biomedical Science Fields

Beyond agricultural activity, N-doped biochar has received significant attention in biomedical applications due to its biocompatibility, antimicrobial properties, and photothermal efficiency [152]. It demonstrates superior antimicrobial activity against pathogens like *Escherichia coli* and Gram-positive *Staphylococcus aureus*, positioning it as a potential alternative to traditional antibiotics [153]. Additionally, N-doped carbon-based materials serve as effective photosensitizing agents in photodynamic therapy (PDT), generating reactive oxygen species (ROS) to target and destroy cancer cells [154]. These materials are also used in drug delivery systems, showcasing their multifunctional potential in medical science [152]. This offers an informative opportunity to explore SMS-derived biochar in advanced biomedical applications.

## 9. Challenges and Limitations of SMS-Derived Biochar Utilization

Carbonization, particularly through pyrolysis, has gained significant attention in recent years for its potential applications in agricultural and environmental contexts [13]. SMS is a distinctive feedstock for carbonization due to its rich content of amino acids and small organic molecules, which gives carbonized SMS, or SMS-derived biochar, unique properties [35]. SMS-derived biochar has shown promising potential, with applications as a fertilizer and even as a growth medium for further mushroom cultivation [13,20,155]. However, despite advances in its use as an adsorbent and fertilizer, the role of carbonized SMS in soil improvement remains underexplored, and research into its use as biochar is still growing [13]. Additionally, biochar-induced dust emissions pose potential long-term health risks to users, particularly in agricultural applications. Developing alternative biochar forms, such as granules, pellets, dust-free types, and liquid biochar fertilizers, could offer more convenient, effective, and lower-risk solutions. Moreover, a study by Horn et al. [156] indicated that the excessive use of biochar will not only impact the environment but also affect the composition of soil matter, potentially affecting the soil’s natural functions.

The limited studies available highlight the need to investigate the effects of different SMS types and pyrolysis temperatures on the biochar’s characteristics and its adsorption capacity for various heavy metals [49]. Many studies have demonstrated that varying these parameters may influence SMS-derived biochar properties, especially its ability to retain essential nutrients and remove contaminants from the soil [12,20,49]. Furthermore, current methods for creating SMS-derived biochar often involve complex and expensive laboratory techniques, which restrict their feasibility for large-scale applications. For SMS-derived biochar to be commercially viable, studies are needed to streamline and optimize the pyrolysis of agricultural wastes like SMS to produce biochar with high adsorption capabilities and practical utility in soil amelioration [46]. This emerging research direction holds promise for further enhancing the ecological and economic value of SMS-derived biochar in sustainable agricultural systems.

## 10. Future Prospects and Research Opportunities

Future research on SMS-derived biochar opens up promising avenues for enhancing agricultural and environmental sustainability. One interesting application is in precision agriculture, where biochar can support soil improvements, controlled-release fertilizers, and advanced water management systems to boost resource efficiency and crop performance. By integrating biochar into modern soil management techniques, farmers can increase nutrient efficiency, reduce input waste, and decrease their use of synthetic fertilizers [8,16,35].

Improving SMS-derived biochar quality and functionality through modified production conditions, chemical enhancements, or blends with other materials is another promising approach, potentially increasing nutrient retention, stability, and pollutant removal efficiency. For example, pyrolyzing biochar at temperatures above 500 °C can improve its capacity to handle multiple contaminants, while acid treatments may enhance pollutant absorption [157]. These modifications can expand the application of biochar beyond soil enhancement, making it a viable solution for water filtration, heavy metal remediation, and carbon sequestration.

Beyond technical advancements, policy support and regulatory frameworks are also crucial to promoting sustainable biochar practices. Government incentives, carbon credit programs, and waste management regulations can make biochar a more economically viable solution, furthering its role in carbon sequestration, waste recycling, soil health, and reducing greenhouse gas emissions in agriculture systems [74,146]. Ultimately, these advancements in SMS-derived biochar could transform it into a highly valuable tool for sustainable agricultural management and environmental conservation. Achieving these goals will require collaboration between researchers, industry leaders, and policymakers to drive innovation and implementation [1].

Importantly, growing biochar technology requires a comprehensive and cooperative strategy that integrates industry-driven innovations, scientific findings, and supportive requirements to optimize its potential. Continuous research, strategic partnerships, and well-defined production standards will be crucial in realizing the full potential of SMS-derived biochar. With sustained efforts in innovation and implementation, SMS-derived biochar can potentially become an important part of sustainable agriculture and environmental resilience and contribute to a circular economy.

## 11. Conclusions

The unique physicochemical properties of SMS, including its lignocellulosic composition, high nutritional profile, and organic matter richness, make it a perfect substrate for biochar production. SMS-derived biochar offers a sustainable solution to numerous challenges in modern agricultural systems. This extensive review discusses the multifaceted benefits of SMS-derived biochar, from its composition and production processes to its applications in soil enhancement, plant growth, water treatment, pest management, and mushroom cultivation. SMS-derived biochar demonstrates superior characteristics, such as increased porosity, nutrient retention, and carbon stability, making it highly effective in improving soil fertility, water retention, and microbial ecosystems and in sequestering carbon to mitigate climate change. Economically, SMS-derived biochar offers a viable alternative to other biochars due to the abundance and low cost of its precursor. Its integration into agricultural systems reduces dependence on synthetic fertilizers, enhancing resource efficiency for both smallholder and industrial farming. Despite its broad potential, challenges such as feedstock variability, diverse pyrolysis methods, and the need for long-term environmental impact assessments remain. Future research should focus on standardizing production methods, exploring innovative applications, studying regeneration techniques, and developing policies to integrate SMS-derived biochar into sustainable agricultural frameworks. Overall, SMS-derived biochar stands as a key innovation in sustainable agriculture systems, aligning with ecological conservation, resource efficiency, and sustainable development goals. It also provides a viable framework for promoting green agricultural practices while contributing to global environmental flexibility.

## Figures and Tables

**Table 1:** The physicochemical properties of SMS remains from each mushroom production.

Properties			Spent Mushroom Substrates					
	*Agaricus bisporus*	*Coprinus comatus*	*Flammulina velutipes*	*Ganoderma lucidium*	*Hericium erinaceus*	*Lentinula edodes*	*Pleurotus ostreatus*	*Tremella fuciformis*
EC (dS m^−1^)	4.83–6.60	0.88	-	1.92	1.08	1.96	0.89–4.01	-
pH	5.02–8.05	5.80	5.83	7.10	4.24	3.79–4.33	5.54–6.49	6.12
OM (%)	66.98	-	-	58.97	90.09	92.73	61.17–91.87	-
OC (%)	23.40–54.30	-	41.24	34.20–44.82	52.25	38.96–53.78	32.61–53.29	40
C/N ratio	11.50–25.43	-	29.88	27.33–74.35	92.60	26.69–40.95	19.00–79.31	19.51
Total N (%)	0.92–2.72	-	1.38	0.46–1.64	0.57	1.12–1.46	0.56–1.72	2.05
Total P (%)	0.21–0.84	-	0.04	0.09–0.36	0.10	0.02–0.07	0.05–0.77	0.03
Total K (%)	0.43–2.40	0.85	0.02	0.48–1.03	0.35	0.10–0.21	0.23–1.90	0.04
Ca (%)	4.70–10.80	5.15	-	2.66–10.92	0.84	0.52	0.68–10.44	-
Mg (%)	0.60–0.91	0.30	-	0.24–0.28	0.18	0.45	0.09–0.51	-
S (%)	-	-	0.7	-	-	0.91	0.20	1.60
Fe (mg·kg^−1^)	174.27–920	-	-	38.27–60	27.93	21.40	7.60–30.80	-
Mn (mg·kg^−1^)	241.20–1600	-	-	100–291.80	282.80	225.40	134.87–225.93	-
Zn (mg·kg^−1^)	136.41–220	-	-	37.47	26.00	56.47	28.20–49.20	-
Cu (mg·kg^−1^)	50.70–79.67	-	-	14.93	11.60	25	10.80–11.60	-
Na (mg·kg^−1^)	600–6693.33	1900	-	886.67	660	1100	433.33–1233.33	-
WHC (%)	277.5	-	-	231.39	324.10	366.30	347.31–699.68	-
References	[16,23,24,25]	[13]	[7]	[24,30]	[24]	[7,12,24]	[20,24,26]	[7]

Note: EC refers to electrical conductivity; C/N represents carbon-to-nitrogen ratio; and WHC refers to water-holding capacity.

**Table 2:** The physicochemical properties of SMS-derived biochar from each mushroom species after the pyrolysis process.

Properties	SMS-Derived Biochar							
	*Agaricus bisporus*	*Coprinus comatus*	*Flammulina velutipes*	*Ganoderma lucidum*	*Lentinula edodes*	*Pleurotus* sp.	*Pleurotus ostreatus*	*Tremella fuciformis*
EC (dS m^−1^)	2.09	3.54	-	-	-	2.81	-	-
pH	8.10	10.05	9.84–11.64	7.78–11.38	6.49–12.07	9.27	9.58–12.31	11.32–11.63
C (%)	62.06	-	54.85–58.61	53.23–70.72	49.44–58.65	50	37.49–69.40	52.60–54.32
C/N ratio	8.67	-	33.9–47.44	27.30–27.73	21.64–24.47	172.41	53.40–85.20	25.89–29.30
CEC (cmol kg^−1^)	-	-	25.71–32.29	-	19.37–27.15	-	-	26.71–28.98
Total N (%)	-	-	1.16–1.67	1.95–2.55	1.09–2.71	-	0.44–1.30	1.80–2.07
Total P (%)	-	-	0.11–0.12	0.71–0.95	0.03–0.06	0.58	3.80	0.06–0.07
Total K (%)	-	1.19	0.08–0.10	0.84–1.26	0.11–1.25	1.39	-	0.12–0.15
Ca (%)	-	9.69	-	3.75–8.94	7.48–11.35	13.36	0.40	-
Mg (%)	-	0.81	-	0.50–0.72	1.09–1.12	1.70	-	-
S (%)	-	-	1.32–1.60	-	0.04–0.49	1.01	-	0.07–0.14
Fe (mg·kg^−1^)	3500	-	-	1500–2500	-	1895	24000	-
Mn (mg·kg^−1^)	1200	-	-	10–20	-	33	-	-
Zn (mg·kg^−1^)	1000	-	-	-	-	84	-	-
Cu (mg·kg^−1^)	-			-	-	10	-	-
Na (mg·kg^−1^)	-	1110	-	290–660	520–920	500	-	-
SSA (m^2^ g^−1^)	5.10	-	148.3–210.57	7.18–105.04	3.09–73.56		17.5–1095	24.47–51.73
PS (nm)	0.92	-	-	2–50	2–50	-	2.75–21.8	-
References	[16]	[13]	[7]	[30,46]	[7,12,33,46]	[36]	[20,33,48]	[7]

**Table 3:** Overview of the SMS-derived biochar’s adsorption capabilities for the removal of heavy metals following modifications and summaries from Madzin et al. [15] and Jiang et al. [79].

Sources of SMS-Derived Biochar	Adsorption Capacity of Heavy Metals (mg·g^−1^)					References
	Cadmium	Chromium	Copper	Lead	Zinc	
Spent mushroom substrate from *A. bisporus*	64.8	-	68.1	-	55.2	[47,80]
Spent mushroom substrate from *A. auricula*	114.6	118.0	-	-	-	[79]
Spent mushroom substrate from *L. edodes*	-	-	-	398	-	[33]
Spent mushroom substrate from *P. ostreatus*	55.95	-	-	326	-	[33,77]
Spent mushroom substrate from *G. lucidum*	23.81–75.82	-	-	141.59–262.76	-	[30]
Spent mushroom compost	-	-	52.6–364	564	332	[49,81]

**Table 4:** The performance comparison of SMS-derived biochar to other biochars, via information modified and summarized from Leng et al. [83], Jatuwong et al. [88], Bolan et al. [99], Tomczyk et al. [119], Ravindiran et al. [120], Wijitkosum, [121], and Ayaz et al. [122].

Biochar Types	pH	C/N Ratio	OC (%)	N (%)	P (%)	K (%)	Ca (%)	Mg (%)	S (%)	Fe mg·kg^−1^	Mn mg·kg^−1^	Zn mg·kg^−1^	Cu mg·kg^−1^	SSA (m^2^·g^−1^)	PS (nm)
SMS-derived biochars	6.5–12.3	26.2–85.2	37.5–70.7	0.44–2.7	0.03–3.8	0.08–1.4	0.4–13.4	0.5–1.7	0.04–1.6	1500–24,000	10–1200	84–1000	10	3.1–1095	0.9–50
Acacia tree	-	5.68–86	51.6–56.8	0.6–1	0.1–1.14	0.71	0.27–0.39	0.001–0.24	-	-	-	-	-	-	-
Apple wood chips	-	117.5–122.7	67–73.6	0.57–0.6	0.00400.18	0.6	2.42	0.32	-	5745	91.5	37.3	9.9	-	-
Bagasse	6.6–9.7	118	71	0.6	0.08	0.43	1.2	0.21	0.03	4800	300	400	15	259	-
Bamboo wood	5.2–8.83	95	76	0.8	0.44	3.5	0.05	0.14	0.08	70	40	6400	15	29.69–369.59	-
Castor stalk	-	16.3–43.8	61.3–78.4	1.4–4.8	0.54–2.98	0.4–3.48	0.8–8.89	0.43–1.42	-	210–4332	35–294	27.2–236	6.8–55	-	-
Cashew wood residues	-	-	-	0.9	0.01	0.13	-	-	-	185	32.3	18.45	10.2	-	-
Chicken manure	8.1–11.7	12.4–13.2	23.7–34.6	1.8–2.8	1.9–3	4.2–6	2–3	2.1–3.8	-	-	-	-	-	-	-
Cocao shell	-	21.7	36.8	1.7	0.59	4.05	2.92	1.47	-	-	-	-	-	-	-
Coconut shell	-	256.7	77	0.3	0.7	0.71	0.04	0.05	0.02	100	20	10	15	613–626.8	0.42–2.85
Coffee waste	9.7–10.05	112.9	79	0.7	0.03	0.35	0.4	0.08	0.025	150	40	45	15	-	-
Conocarpus wastes	-	91.7	64.2	0.7	0.084	0.038	4.34	0.343	2.28	-	-	-	-	-	-
Corn cob	7.7– 10.1	18.6–65.9	79.1–80	1.2–4.3	0.31	1.8	0.14	0.16	0.11	800	50	80	20	53.7–56.9	59.66
Corn stover	7.2–10	50.9–53.7	50.9–69.8	1–1.3	0.14–0.23	1.71–2.88	0.03–10.65	0.10–0.59	0.07	-	142	132	963	3.1–712	-
Cotton stalk	-	42.3	71.9	1.7	1.22	0.4	4.33	1.12	-	4332	161	27.2	47.2	-	-
Cow manure	9.20	46.6–224	33.6–60.6	0.15–1.3	0.2–0.8	0.01–0.26	0.04–0.94	0.03–0.4	0.11	376	137	162	-	2.5–8	-
Dairy manure	6.8–10.5	37.8–186	55.8–56.7	0.3–1.5	1.0–1.69	1.43–2.31	2.67–4.48	1.22–2.06	0.11–0.15	36,700–44,800	525–867	363–363	99–163	1.6–186.5	-
Digested dairy manure	-	247.5–250.9	57.7–59.4	0.23–0.24	0.65–0.83	1.49–1.66	2.65–2.67	0.85–0.97	0.27–0.29	1656–2356	145–191	131–200	-	-	-
Eucalypt green waste	5.9–9.7	203.8–238.1	61.9–81.5	0.26–0.4	0.005–0.23	0.03–1.1	0.05–4.3	0.04–1.04	0.014–0.08	30–7000	180–1400	20–200	5.0–12	-	-
Fall yard waste	-	55.2	60.7	1.1	0.21	1.08	5.46	0.36	0.1	1504	555	70	-	-	-
Food waste	-	14.2	52.4	3.7	0.05	1.46	5.17	0.53	0.08	4431	179	39	-	-	-
Grass clippings	-	10.9	53.5	4.9	1.2	6.13	2.06	0.63	0.63	1557	360	150	-	-	-
Indian pine		65.7	72.3	1.1	0.11	0.04	0.17	0.07	0.63	-	-	-	-	-	-
Lantana weed	-	129.8	77.9	0.6	1.33	0.06	0.12	0.1	0.08	-	-	-	-	-	-
Lemon peel	-	28.7	66	2.3	0.27	2.9	2.1	2.6	0.06	90	30	20	25	-	-
Macadamia shell	-	195	78	0.4	0.24	2.19	0.37	0.17	-	1211	-	-	-	-	-
Oil palm fruit	-	46	69	1.5	0.51	3.02	1.4	0.64	0.14	200	200	120	55	-	-
Pearl millet stover	10.6	26.9–48.5	53.3–59.1	1.1–2.2	0.05–1.6	2.24–2.95	0.04–1.47	0.19–1.06	-	-	-	-	-	-	-
Poultry litter	8.7–10.3	19.2	38.3	2	0.9	1	2.5	0.3	-	2695	265	238	57	1–50.9	-
Pruning fruit waste	10.4–10.8	86.4–135	67.5–77.8	0.5–0.9	0.23–0.27	1.39	2.5	2.87	0.005	0.033	0.008	0.01	0.09	92	-
Rice husk	7.5–9.2	76.7	46	0.6	0.3	0.85	0.13	0.17	0.009	200	450	300	20	51.39–525	31.63
Rice straw	6.7–10.8	38.8–73.8	44.3–46.6	0.6–1.2	0.019–0.092	2.41–2.82	0.058	0.2	0.25	-	-	-	-	772.3	2.18
Sewage sludge	-	15.7	26.6	1.7	0.52	6.57	0.64	-	-	22,100	450	1520	380	408	5.21
Sugar maple waste	-	266.7	80	0.3	0.02	0.32	0.5	0.06	0.09	49.7	368	23.9	5	-	-
Swine manure	7.8–11	26.3–30.5	54.9–57.9	1.8–2.2	0.98–1.55	1.62–3.53	2.03–2.89	1.57–2.13	0.02–0.04	-	-	-	-	4.9–15.9	-
Swine solids	8.4–9.5	14.7–17	44.1–51.5	2.6–3.5	3.89–5.9	1.78–2.57	3.91–6.15	2.44–3.69	0.80–0.85	48,400–74,800	1453–2440	3181–4981	1538–2446	-	-
Tamarind wood	-	112.9	86	0.7	0.04	0.53	1	0.06	0.03	5	5	10	4	-	-
Turkey litter	9.9	12–23.6	44.8–49.3	1.9–4.1	2.68–3.63	4.01–5.59	4.04–5.61	0.85–1.24	0.41–0.55	27,800–36,500	710–986	690–909	535–762	-	-
Wheat straw	6.7–11	46.8–50.4	55.4–60.8	1.1–1.3	0.012–0.041	2.06–3.22	0.030–0.047	0.01–0.02	-	-	-	-	-	4.5–246.2	0.975
Willow wood waste	-	118.7	47.5	0.4	-	-	-	-	0.19	0.05	110	83.5	2.55	11.4–840.6	0.545

**Table 5:** A comparison between the unit cost of SMS-derived biochar and biochar made from various kinds of biomass in different locations using summarized information collected by Hu et al. [14], Alhashimi and Aktas, [128], and Zhang et al. [129].

Biomass Types	Locations	$/Ton
SMS	Chongqing, China	117.5
Bamboo	Alabama, USA	7000
Chicken manure	Republic of Korea	1300
Coconut shell	USA	800
Coppiced hardwoods	UK	1600
Corn debris, manure, and forestry debris	Idaho, USA	1500–2000
Hardwood	Australia	2300
Mixed hardwood and softwood biochar	Central Canada	1000
*Oiltea camellia* shell	USA	670
Pinewood	Missouri, USA	900–1900
Pine tree-derived organic biomass	Oregon, USA	8300
Sewage sludge	USA	560–1000
Softwood chips	California, USA	3500
Various timbers mixed with softwoods	Vermont, USA	7200
Switchgrass	USA	5490
Tree branches	Kansas, USA	11,000
Virgin wood feedstock	Massachusetts, USA	17,800
Water oak wood	USA	770

## Data Availability

Data are contained within the article.
